# Knowledge of novel coronavirus disease (COVID-19) among dental surgeons of Nepal: a nationwide study

**DOI:** 10.1186/s12879-020-05620-4

**Published:** 2020-11-23

**Authors:** Mukesh Kumar Sah, Abanish Singh, Raj Kumar Sangroula

**Affiliations:** 1 Department of Emergency Medicine and General Practice, Patan Academy of Health Sciences, Lalitpur, Bagmati Province Nepal; 2 Narayani Hospital, Birgunj, Province 2 Nepal; 3 Nepal Public Health Research and Development Center, Shankhamul, New Baneshor, Kathmandu, Bagmati Province Nepal

**Keywords:** Case management, COVID-19, Dental surgeons, Knowledge

## Abstract

**Background:**

COVID-19 is an emerging respiratory disease caused by a novel coronavirus. There is not much evidence assessing the knowledge of dental surgeons regarding COVID-19. This study was conducted to assess the knowledge of COVID-19 among dental surgeons of Nepal.

**Methods:**

A web-based cross-sectional study was conducted among registered dental surgeons of Nepal. Ethical approval was obtained. Consent was taken, and knowledge on COVID-19 was assessed via a pre-tested structured questionnaire using Google form. The form was emailed to the participants. Descriptive analysis was performed using frequency, percentage, median and inter-quartile range. Man-Whitney test and Kruskal-Wallis tests were carried out to see the difference in knowledge score. *P*-value < 0.05 was considered statistically significant.

**Results:**

Total 227 dental surgeons responded to the questionnaire (male: 46.4%; female: 53.7%). Almost two-third ( 65.2% ) of the respondents were B.D.S. (Bachelor of Dental Surgery) graduates. Only 29.1% worked in the government hospitals. 17.6% were currently involved in COVID-19 management. Of the participants, 87.7% knew about the condition of the requirement of Personal Protective Equipment (PPE) but only 29.1% could correctly answer the framed question for PPE. The median knowledge score calculated was 14.0 (8.0–18.0). The bivariate analysis showed a statistically significant difference in knowledge score among the age group ≥30 years and < 30 years (*p* = 0.013); M.D.S. (Master of Dental Surgery) graduate and B.D.S. graduate (0.041); dental surgeons of government healthcare facilities and other healthcare facilities (*p* <  0.001); dental surgeons of COVID-19 centers and non-COVID-19 centers (0.002).

**Conclusion:**

The dental surgeons of Nepal have a good knowledge of COVID-19, and they can be utilized for assisting in the management of COVID-19 cases in Nepal.

**Supplementary Information:**

The online version contains supplementary material available at 10.1186/s12879-020-05620-4.

## Background

Coronavirus disease (COVID-19) is an emerging respiratory disease caused by a novel coronavirus [1]. It was first reported to World Health Organization (WHO) country office in China from Wuhan city, China on 31 December 2019 [1]. The highly infectious disease has infected more than 6 million people with the reported deaths of more than 378,000 and spreading to 213 countries worldwide [2]. WHO declared this outbreak as a Public Health Emergency of International concern on 30 January 2020 [2].

COVID-19 is largely spread by human-to-human transmission through droplet infection with some cases of feco-oral and direct transmission [3]. The incubation period is 2–14 days [3]. The main clinical symptoms include fever, dry cough, fatigue, myalgia and dyspnea. There is no antiviral treatment or vaccine recommended for COVID-19. Hence, WHO, along with national and international health agencies, has highly recommended preventive strategies like frequent hand washing, social distancing, and lockdown. Despite such stringent global efforts, the numbers of cases are increasing day by day. This has led to increased load among medical doctors and nurses to combat this pandemic. Now, it is seen that the available health facilities and medical doctors are running short and dental doctors are being called upon to deal with the pandemic [4, 5].

The doctor-patient ratio of Nepal is 0·17 per 1000 population—substantially less than the WHO recommendation of 2·3 doctors per 1000 population [6].The number of COVID-19 cases are increasing in Nepal as well. As of August 20, 2020, the official data of Nepal shows 29,645 people infected with COVID-19 and 126 registered deaths due to COVID-19 [7]. Now, many health care workers are already infected with COVID-19 leading to a shortage of human resources to combat COVID-19 that has led for an emergency call for recruitment of health care professionals [8]. If the medical doctor runs short in Nepal to deal with COVID-19, then dental surgeons might be of significant support to deal with this pandemic. Dental surgeons are the most efficiently trained doctors regarding the medical diseases after medical doctors as they share a common curriculum of 3 years out of the 5.5 years. There are many online training manuals by WHO and different health agencies to prepare all health care professionals for COVID-19 [9].There are few evidences assessing the knowledge of dental surgeons regarding COVID-19 [10, 11]. Hence, this study was conducted to assess the knowledge of COVID-19 among dental surgeons of Nepal.

## Methods

### Study design, study population and sample size

A web-based quantitative descriptive cross-sectional study was carried out from 10 March to 25 March, 2020. This study considered (95% CI) and 95% power to estimate the sample size. For this purpose, 65.7% (p) was considered from a study [12] conducted among health workers on knowledge and perception related to COVID 19, and the calculated sample size was 200. A structured questionnaire using Google forms was emailed to all the Nepal Medical Council registered dental surgeons. Emails were obtained from the Nepal Dental Association dentist directory. A total of 227 registered dentists filled the Google forms by May 25, 2020, which were analyzed.

### Data collection procedures and ethical approval

A pre-tested structured questionnaire using Google form was emailed to collect data from registered dental surgeons of Nepal. Ethical approval was taken from the Ethical Review Board (ERB) of Nepal Health Research Council (Reg. no.: 2383). The Google forms emailed had informed consent form with details of the study. A reminder email was sent to all the previously emailed dental surgeons after a week of the first email. Confidentiality of the information was ensured by maintaining privacy.

### Study variables

#### Socio-demographic variables

Age of the participants was categorized as less than 30 and more than or equal to 30. The gender of the participants was categorized as male and female. The degree of the dental surgeons was categorized as Bachelor of Dental Surgery (B.D.S.) and Master of Dental Surgery (M.D.S.). The working province of the participants included all the seven provinces of Nepal in which Karnali and Sudurpaschim Province were merged for bivariate analysis. Type of hospital/ health center where the dental surgeons worked was categorized as Government, Private clinic, Private hospital, and semi-government. The level of hospital/health center was categorized as Primary, Secondary and Tertiary levels. Working hospitals of the dental surgeons was categorized into COVID-19 center and non-COVID-19 center. Involvement in COVID-19 management was categorized into “Yes” and “No”. Received training on COVID-19 management was categorized into “Yes” and “No”.

#### Knowledge on COVID-19

For assessment of knowledge on COVID-19 among dental surgeons of Nepal, a 14-items questionnaire tool was developed from the published literatures [10–12]. Face and content validation of tool was done by the researchers. Further, the tool was pre-tested among 25 dental surgeons to check for reliability. These 25 dental surgeons were not included in the main study. The tool included questions related to the origin of Coronavirus, case definition of COVID-19, contact and contact tracing, condition of requirement of PPE, principles of Rapid Diagnostic Test (RDT) and Reverse Transcription Polymerase Chain Reaction (RT-PCR) test, sample collection and transportation, preventive measures (quarantine and isolation), waste management, clinical features of COVID-19, roles and responsibility of different level of hospitals for COVID-19 management and public awareness for the prevention of COVID-19. The cumulative score of the knowledge related questions ranged from 0 to 18 (Table 1).

**Table 1:** shows the score related to each component

Knowledge components	Knowledge score
Case definition of COVID-19	3
Definition of contact and contact tracing	1
Transmission of COVID-19	1
Need and use of PPE	1
Test for COVID-19	1
Sample collection and transportation	1
Preventive measures for COVID-19	1
Waste management	1
Clinical features	2
Epidemiological patterns	1
Government assigned hospital levels in Nepal for management of COVID-19	1
Public awareness for prevention of COVID-19	1
**Overall knowledge score on COVID-19**	**18**

### Statistical analysis

All the responses from Google forms were exported to Microsoft Excel 2016 and were exported to SPSS version 23 (SPSS, Inc., Chicago, IL, USA) software. Descriptive analysis was performed using frequency, percentage, median and inter-quartile range. After the normality test of the knowledge score, it was found that the overall knowledge score was non-normal (skewed). Man-Whitney test (for variables with two categories) and Kruskal-Wallis test (for variables with more than two categories) were carried out to see the difference in knowledge score between different socio-demographic variables. *P*-value < 0.05 was considered statistically significant.

## Results

This was a web-based survey conducted to assess the knowledge of COVID-19 among dental surgeons of Nepal. Total 227 dental surgeons responded to the questionnaire (male: 46.4%; female: 53.7%). Almost two-third (65.2%) of the respondents were B.D.S. graduates. More than 80% of the respondents were from three provinces (Province 1, Province 2, and Bagmati Province). It was seen that only 29.1% of the dental surgeons worked in government hospitals, and more than 50% of the dental surgeons worked in a tertiary level health care facility. More than one-third (35.7%) of the dental surgeons are currently working in a health facility that is a COVID-19 center. It was seen that 17.6% of dental surgeons are currently involved in COVID-19 management. Only 28.2% of dental surgeons received COVID-19 management training, but the majority of them had received online training (71.8%) (Table 2).

**Table 2:** Socio-demographic Characteristics

Characteristics	Dental Surgeons (*n* = 227)	Percentage (%)
**Age**		
< 30 years	128	56.4%
≥ 30 years	99	43.6%
**Gender**		
Male	105	46.3%
Female	122	53.7%
**Education level**		
Bachelor level (B.D.S.)	148	65.2%
Masters level (M.D.S.)	79	34.8%
**Province**		
Province 1	63	27.8%
Province 2	26	11.5%
Bagmati Province	99	43.6%
Gandaki Province	16	7.0%
Province 5	18	7.9%
Karnali + Sudurpaschim Province	5	2.2%
**Type of hospital/ health care center**		
Government	66	29.1%
Private Clinic	58	25.6%
Private Hospital	80	35.2%
Semi-Government (NGO, INGO, Public Private Partnership)	23	10.1%
**Hospital level**		
Primary level	50	22.0%
Secondary level	58	25.6%
Tertiary level	119	52.4%
**Working hospital/healthcare facility**		
COVID-19 Center	81	35.7%
Non-COVID1–19 Center	146	64.3%
Involvement in COVID-19 management		
Yes	40	17.6%
No	187	82.4%
**Ever received COVID-19 management training**		
Yes	64	28.2%
No	163	71.8%
If yes, the type of training received (*n* = 64)		
Web based training	46	71.8%
Workshop/Seminar	18	28.2%

The knowledge of COVID-19 was assessed via a ‘yes-no’ questionnaire followed by a conceptually framed question that checked the accurate knowledge responded to those ‘yes-no’ questionnaires. Almost all dental surgeons were aware of the origin of Coronavirus. 88.5% knew about the case definition of COVID-19, but 79.7% could give the correct answer regarding the same. Of the participants, 87.7% of the dental surgeons knew about the condition of the requirement of Personal Protective Equipment (PPE), but only 29.1% could correctly answer the framed question for PPE. Similarly, 93.8% knew about the preventive measures (quarantine and isolation), but only 59.5% could correctly answer the framed questionnaire (Table 3).

**Table 3:** Knowledge on COVID-19 (*n* = 227)

Knowledge questions with ‘yes’ and ‘no’ responses	Dental Surgeons with “yes” responses (%)	Dental Surgeons with correct responses (%)
Origin of Coronavirus	224 (98.7%)	224 (98.7%)
Case definition of COVID-19	201 (88.5%)	181 (79.7%)
Contact and contact tracing	201 (88.5%)	215 (94.7%)
Condition of requirement of PPE	199 (87.7%)	66 (29.1%)
Principles of RDT and RT-PCR test	167 (73.6%)	178 (78.4%)
Sample collection and transportation	161 (70.9%)	175 (77.1%)
Preventive measures (quarantine and isolation)	213 (93.8%)	135 (59.5%)
Waste management	109 (48.0%)	126 (55.5%)
Clinical features of COVID-19	224 (98.7%)	153 (67.4%)
Roles and responsibility of different level of hospitals for COVID-19 management	130 (57.3%)	143 (63.0%)
Public awareness for prevention of COVID-19	214 (94.3%)	224 (98.7%)
**Overall knowledge score on COVID-19**	**14.0 (8.0–18.0)**	

The knowledge score was also assigned for the correct answer to the questionnaires and the median knowledge score calculated was 14.0 (8.0–18.0).

The bivariate analysis showed that the knowledge score of dental surgeons of age group ≥30 years had better knowledge compared to the age group < 30 years, and the difference was statistically significant (*p* = 0.013). The knowledge score of male dental surgeons was more compared to female dental surgeons, but it was not statistically significant (0.063). M.D.S. trained dental surgeons had more knowledge of COVID-19 compared to B.D.S. trained dental surgeons, which was statistically significant (0.041). The dental surgeons working in government healthcare facilities had better knowledge scores compared to other healthcare facilities, and it was statistically significant (*p* <  0.001). Similarly, the dental surgeons working in COVID-19 centers had higher knowledge compared to non-COVID-19 centers, and the difference was statistically significant (0.002). The dental surgeons involved in the management of COVID-19 patients had better knowledge scores than the ones not involved in COVID-19 management, and it was statistically significant (0.004). But, there was no statistically significant difference between the knowledge score of dental surgeons who received training on COVID-19 and who did not (*p* = 0.853) (Table 4).

**Table 4:** Comparison of knowledge score among different variables

Characteristics	Median (Q1-Q3)	***P***-value
**Age in years**		
< 30	14 (12–15)	0.013*
≥ 30	14 (13–15)	
**Gender**		
Female	13.5 (12–15)	0.063*
Male	14 (13–15)	
**Education level**		
BDS	14 (12–15)	0.041*
MDS	14 (13–15)	
**Province**		
Province 1	14 (13–15)	0.010**
Province 2	15 (13–16)	
Bagmati Province	13 (12–15)	
Gandaki Province	13.5 (12–14.75)	
Province 5	14 (12.75–15)	
Karnali+sudurpaschim Province	15 (14.5–16.5)	
**Type of Healthcare**		
Government	15 (14–16)	< 0.001**
Private	13 (12–15)	
Semi-government	14 (13–15)	
**Level of hospital/healthcare**		
Primary	13.5 (12–15)	0.029**
Secondary	13 (11–15)	
Tertiary	14 (13–15)	
**Working Hospital**		
COVID-Center	14 (13–15.5)	0.002*
Non-COVID Center	13 (12–15)	
**Involvement in COVID-19 management**		
Yes	14.5 (13–16)	0.004*
No	14 (12–15)	
**Training on COVID-19**		
Yes	14 (13–15)	0.853*
No	14 (12–15)	

*Mann-Whitney test; **Kruskal-Wallis test; *p *< 0.05 indicates statistically significant

## Discussions

COVID-19 has imposed a global impact affecting all the sectors and has created a chaotic situation at healthcare facilities. Despite putting a stringent effort by the government like lockdown and promoting preventive measures like frequent hand-washing, use of masks, and social distancing, the numbers of cases are increasing day by day. Globally, the healthcare facilities are running short of trained medical doctors as well. Now in many countries, dental surgeons are being called upon to assist in the management of COVID-19 cases. The preparedness of the healthcare personals to combat any pandemic has been assessed by evaluating the knowledge of the disease [13–15].

This study assessed the knowledge of COVID-19 among the Nepal Medical Council registered dental surgeons. The median knowledge score of 14.0 (8.0–18.0) indicates a good knowledge of COVID-19 among dental surgeons of Nepal which was similar to the study conducted by Kamte et al. [11] The majority of the dental surgeons who responded were B.D.S. graduates (65.2%) and female dental surgeons (53.7%). A similar finding was seen in the survey conducted among Jordanian dentist [16] but was different from the survey conducted among the north Italian dentist [17] where male dentist participated more. More than 80% of the dental surgeons were from province 1, 2, and Bagmati province. The findings were in accordance with the study by Shrestha et al. where it explains the distribution of dental surgeons throughout Nepal [18]. It was seen that 17.6% of dental surgeons were involved in the management of COVID-19 cases. There is no published evidence to explicitly look into the scope for utilization of dental surgeons for the management of COVID-19 patients, but there are pieces of literature that have looked into the knowledge of COVID-19 among dentist [10, 11, 16, 17].

This was a nation-wide study and it was promising to know that almost all the dentists were aware of the origin of COVID-19. The high infectivity of COVID-19 has alarmed to keep the healthcare personals safe. If not so, then the infected asymptomatic healthcare personals might be the carrier and be the medium of transmission. Hence, PPE has been recommended by WHO to be used compulsorily while dealing with suspected/diagnosed cases of COVID-19 [9]. Of the participants, 87.7% of the dental surgeons were aware of the condition of requirement of PPE but only 29.1% could answer the framed question regarding the same. Similarly, 93.8% knew about the preventive measures (quarantine and isolation), but only 59.5% could correctly answer the framed questionnaire. Similar results were demonstrated by Kamte et al. where only 43.7% were sensitized about the WHO guidelines for COVID-19 prevention [11].

In the medical field, an increase in age and experience improves the knowledge of the clinicians. The senior clinicians are mostly postgraduates. They have better knowledge about research, and their practices are mostly evidence-based [19, 20]. It was seen that dental surgeons of age group ≥30 years had better knowledge scores compared to the age group < 30 years (*p* = 0.013). During the postgraduate training, the residents are trained about research and are updated with knowledge based on recent evidences. In contrast, very few institutions are seen to engage their undergraduates in such learning practices [21]. This might be the reason for M.D.S. graduate dental surgeons to have a statistically significant better knowledge score compared to B.D.S. graduates (*p* = 0.041). These findings were similar to those of Kamte et al. [11] and Gupta et al. [22]. The dental surgeons (29.1%) working in government healthcare facilities had a better knowledge score of COVID-19 compared to other healthcare facilities, and it was statistically significant (*p* < 0.001). It was a similar situation among Jordanian dentists where 22.8% were working in public service whereas it was only 6.82% of north Italian dentist associated with National health System [16, 17]. Similarly, the dental surgeons working in COVID-19 centers had higher knowledge compared to non-COVID-19 centers, and the difference was statistically significant (0.002). This might be due to the reason that currently in Nepal, only government healthcare facilities are used to treat COVID-19 cases, and the healthcare personals are oriented and trained before the management of COVID-19 cases.

Traditionally seminar, workshops, hands-on training with face to face interactive sessions was used for regular updates on recent evidence. The outbreak of COVID-19 has limited all the mass gathering and social events to decrease the transmission rates. The recent evidence suggests that online training can be of similar significance compared to on-site training, but further exploration is needed to establish it as fact [23, 24]. It was seen that 28.2% of the dental surgeons had received training COVID-19. But, there was no statistically significant difference between the knowledge score of dental surgeons who received training on COVID-19 and who did not (*p* = 0.853).

The strength of the study is that, it was a nation-wide study where the participating dental surgeons were from all the provinces. The calculated sample size was obtained, and the results can be generalized throughout Nepal. This study had some limitations, the web-based cross-sectional nature of the study design that is prone to selection bias and information bias on the respondent’s side.

## Conclusion

In conclusion, the dental surgeons of Nepal have a good knowledge of COVID-19. Based on the finding of this study, dental surgeons can be utilized for assisting in the management of COVID-19 cases when needed. But, they are to be oriented and trained before, about management protocols of COVID-19.

## Supplementary Information

**Additional file 1.** Questionnaire.

## Data Availability

The data supporting the findings of this article are available from the corresponding author.

## Abbreviations

COVID-19: Coronavirus Disease − 19
B.D.S.: Bachelor of Dental Surgery
M.D.S.: Master of Dental Surgery
PPE: Personal Protective Equipment
CI: Confidence Interval
ERB: Ethical Review Board
RDT: Rapid Diagnostic Test
RT-PCR: Reverse Transcription – Polymeric Chain Reactions
