# Supplementary material for: Knowledge of novel coronavirus disease (COVID-19) among dental surgeons of Nepal: a nationwide study
Source: BMC Infect Dis. 2020 Nov 23;20:871. doi: 10.1186/s12879-020-05620-4 (PMC7681182; doi:10.1186/s12879-020-05620-4)
Supplement: Supplementary file 1 — Additional file 1. Questionnaire. [file 12879_2020_5620_MOESM1_ESM.docx]

**Questionnaire**

Name:

Age: Gender:

1. Degree:
   1. BDS
   2. MDS
2. Province of the hospital/Health Center where you work:
   1. Province 1
   2. Province 2
   3. Bagmati Gandaki
   4. Province 5
   5. Karnali
   6. Sudurpaschim
3. Type of hospital/health center where you work:
   1. Government
   2. Private Hospital
   3. Private Clinic
   4. Semi-Government (NGO, INGO, Public Private Partnership)
4. Level of hospital/health care center where you work:
   - - 1. Primary
       2. Secondary
       3. Tertiary
5. Is your hospital/health care center is the center for COVID-19 treatment?
   - - 1. Yes
       2. No
6. Are you involved in COVID-19 management till now?
   - - 1. Yes
       2. No
7. Have you ever participated in COVID-19 management training?
   - - 1. Yes
       2. No

If yes, mention the type of training.

- 1. Web based training
  2. Workshop/seminars

1. The COVID-19 virus is known to originate from
   1. Pigs
   2. Bats
   3. Chickens
   4. Mosquitos
2. Regarding the case definitions for COVID-19, which of the following is known to you (you can chose more than one options)?
   - - 1. Suspected case
       2. Probable Case
       3. Confirmed Case
       4. All of the above
3. Do you know definition of contact in case of COVID-19?
   - - 1. Yes
       2. No
4. Do you know about contact tracing?
   - - 1. Yes
       2. No
5. How the corona virus is transmitted?
   - - 1. Droplet
       2. Faeco-oral
       3. Sexual
       4. Via vector
6. Do you know the condition of requirement of full or part of PPE?
   - - 1. Yes
       2. No
7. Is full PPE required to see all patients who come to the hospital?
   - - 1. Yes
       2. No
8. Do you know about RDT and RT PCR tests for COVID-19?
   - - 1. Yes
       2. No
9. Do you know the principles of RDT and RT PCR test for COVID-19?
   - - 1. Yes
       2. No
10. Do you know the sample collecting methods and its transportation in a case of COVID-19?
    - - 1. Yes
        2. No
11. Do you know when to quarantine and when to isolate a person?
    - - 1. Yes
        2. No
12. Do you know the method of disinfection of the waste from COVID-19 infected area in a hospital/health care facility?
    - - 1. Yes
        2. No
13. Do you know the sign, symptoms and complications of COVID-19?
    - - 1. Yes
        2. No
14. Now in Nepal, in which stage of COVID-19 epidemiologic pattern is seen?
    - - 1. Sporadic
        2. Cluster
        3. Community
        4. Disaster
15. Do you know the role and responsibilities of different level of hospitals assigned for COVID 19 by government of Nepal?
    - - 1. Yes
        2. No
16. Do you know the methods of prevention that should be explained to the public and to the health personnel?
    - - 1. Yes
        2. No
17. A 20 year female who visited US two week ago, presented in dental OPD for toothache. She has no fever, dry Cough, shortness of breath or headache. As she has recently visited US, as per government protocol you have sent her sample for RT PCR test for COVID-19. The test is positive. What is your diagnosis?
    1. Suspected case of COVID
    2. Probable case of COVID
    3. Definitive/Confirmed case of COVID
    4. Contact COVID
18. A 27 year gentleman arrived Nepal from Italy two weeks ago via a flight of Nepal Airlines. Unfortunately he develops fever, cough and shortness of breath and then he visited Teku Hospital where he was diagnosed as a case of COVID-19 and he was kept in the isolation ward. Now Nepal government wants to test all other person who has travelled via the same flight of the Nepal Airlines. The method of tracing the other persons is.
    - - 1. Contact tracing
        2. Random Sampling
        3. Stratified Sampling
        4. Snowball Sampling
19. In a hospital there is separate fever clinic where all fever patients go. The dental department of the hospital asks for full PPE while examining the patient in the dental clinic, what will be your recommendation?
    - - 1. Full PPE
        2. Head Cover, Goggles, Mask, Gloves
        3. Surgical Mask and Gloves
        4. N95 mask and Gloves
20. Acute COVID-19 infection is diagnosed by
    - - 1. RDT for IgG
        2. RT PCR
        3. Blood C/S
        4. Stool C/S
21. RDT is usually positive after how many days of infection?
    - - 1. 1 day
        2. 2 days
        3. 3 Day
        4. 7 Days
22. Before transporting to Teku Hospital how will you seal the nasopharyngeal swab that is collected from a suspected case of COVID-19?
    - - 1. VTM, Zip Lock Bag, Ice box
        2. VTM, Zip Lock Bag
        3. VTM
        4. VTM, Ice Box
23. You have taken round in isolation ward for toothache of a patient who is already diagnosed COVID-19. How will you disinfect the PPE that have used after visiting a COVID patient in isolation ward?
    - - 1. Pack in biohazard bag and throw it
        2. Pack in biohazard bag and autoclave it
        3. Disinfect with Chorine
        4. Burrier in soil
24. A 37-year male who have recently visited Spain presented in dental clinic with complain of toothache. The dental surgeon treated him. 2 days later the patient was diagnosed as confirmed case of COVID 19. What is your recommendation for treating dental surgeon?
    1. Isolate him
    2. Ask him to stay in quarantine
    3. Get him admitted in hospital
    4. Treat him as a COVID 19 patient
25. Besides respiratory system which organs are affected in severe case of COVID 19?
    - - 1. Heart
        2. Kidney
        3. Liver
        4. Both a and b
26. According to government, BPKIHS is which level of hospital for COVID 19 treatment?
    - - 1. Level I
        2. Level II
        3. Level III
        4. Level IV
27. Which of the following recommendation you give for public to be safe from COVID 19 infection?
    - - 1. Wash hands frequently or sanitize
        2. Stay at Home
        3. Maintain Social Distancing
        4. All of the above

End
